# Supervision Effects on Negative Affect and Psychological Distress: Evidence from Social Workers in China

**DOI:** 10.3390/ijerph20031764

**Published:** 2023-01-18

**Authors:** Bin Tu, Chienchung Huang, Sophie Sitar, Yulu Wang

**Affiliations:** 1 Guangdong Research Center for NPO, Guangdong University of Foreign Studies, Guangzhou 510420, China; 2 School of Social Work, Rutgers University, New Brunswick, NJ 08901, USA; 3 Law School, Rutgers University, Newark, NJ 07102, USA; 4 School of Public Administration, Guangdong University of Foreign Studies, Guangzhou 510420, China

**Keywords:** China, job demands, negative affect, psychological distress, social workers, supervision

## Abstract

Supervision is an imperative practice within the social work field. It provides social workers with support systems, ensures that social workers are adhering to professional standards, and protects clients. Research has also shown that quality supervision can improve social workers’ professional capacity and reduce work stress. However, most of this research has been confined to social workers’ experiences within Western countries and has been largely qualitative in nature. Thus, this study aims to examine the experience of 489 social workers based in Guangzhou, China to understand how supervision affects their negative affect and psychological distress. The findings indicate that supervision not only reduces negative affect and psychological distress amongst Chinese social workers, but also is especially effective for social workers with high job demands. When job demands are high, social workers who receive both individual and group supervision also appear to have lower negative affect and psychological distress as compared to social workers who only receive individual supervision. These findings emphasize the significance of supervision as a buffer factor to reduce negative affect and psychological distress amongst Chinese social workers who face high job demands.

## 1. Introduction

### 1.1. Development of Social Work in China

Within the last forty years, social work in China has been steadily on the rise. In the late 1980s, China was nearly void of social workers, but by 2020, the industry had exponentially grown to about 1.5 million social workers in the field [1,2,3]. However, with this rapidly forming professional care industry came major problems. For example, the social work industry had become riddled with high burnout and turnover rates throughout the country [4,5,6,7] because of the steep emotional job demands the work requires [8,9,10,11]. For example, Huang and colleagues found that, of 897 social workers from Chengdu, China, a moderate amount of the participants experienced negative affect and psychological distress. Moreover, they found that participants’ job demands significantly increased negative affect (beta = 0.32, *p* < 0.001) and psychological distress (beta = 0.24, *p* < 0.001) [12]. Thus, Huang and colleagues concluded that increased negative affect and psychological distress amongst social workers can often lead to employees’ negative well-being [12,13,14]. 

The job responsibilities of frontline social workers in China are wide-ranging. Social workers are required to perform client visits and outreach, case management, group work, project planning and execution, community networking, assessment, and training [15,16]. In addition, social workers need to accomplish comprehensive administrative tasks that stem from government-required service-purchasing agreements [17,18,19]. Consequently, social workers frequently work overtime due to the immense needs of clients, projects, and administrative tasks. 

### 1.2. Dangers of Negative Affect and Psychological Distress on Social Work Employees

Negative affect is described as an unpleasant feeling or emotion that can be expressed as guilt, shame, anxiety, fear, irritability, or sadness [20,21]. Negative affect can cause disturbances in an individual’s physical and mental well-being. For example, negative affect can cause emotional dysregulation and psychiatric symptoms [21,22,23], and even reduced job performance [13,24]. The higher the level of negative affect an individual experiences, the more intense negative feelings, such as anxiety and nervousness, will be present [25].

Meanwhile, psychological distress is a state of emotional anguish, due to stress-related events, that can make daily life incredibly difficult [26,27]. Typically, psychological distress is characterized by symptoms of depression, anxiety, or other somatic complaints [27] that can be worsened by perceived stress, especially at work [28]. This type of distress additionally increases an individual’s risk for developing a multitude of behavioral disorders and illnesses, such as mood and anxiety disorders [29,30] as well as increased work problems [29,30,31,32], and even suicidal behavior [33,34]. 

Together, negative affect and psychological distress have significant consequences on an individual’s mental and behavioral well-being, so it is critical that employers understand how supervision policies could mitigate such occurrences for their social workers with high job demands. First, negative affect can anticipate how an individual will perform at work far more accurately than certain personality factors such as neuroticism [25]. Moreover, negative affect has serious influence on job performance because it increases the likelihood of worker withdrawal and job-related injuries while reducing the likelihood of modeling organizational citizenship behavior [25]. Second, psychological distress reduces work performance because it deteriorates overall psychological well-being [28]. According to Wright & Cropanzano [35], individuals with low psychological well-being are less likely to feel happy, especially at work, which can reduce productivity and increase burnout amongst employees. While both of these mental states have serious repercussions within the professional realm, they can each be mitigated by adequate, positive supervision at work. 

### 1.3. Concept and Function of Social Work Supervision

Social work supervision is a process of interpersonal communication between the supervisor and the supervisee that focuses on promoting the development of professional knowledge, skill, and ethical standards within the practice of social work. Moreover, supervisors are able to monitor supervisees’ advanced knowledge and skills and ensure that they are ethically and competently applying their capabilities [36]. Social work supervision also involves administrative, educative, and supportive functions, each working in tandem to ensure that social workers are supported in their work, delivering professional, quality services, and that clients are being protected from harm [36,37,38]. Research on social work supervision has shown that supervision not only improves the quality of services that social workers provide to clients [39,40,41,42,43,44], but that supervision also reduces work stress and increases job satisfaction amongst supervised social workers [45,46,47,48]. Mor Barak et al. performed a meta-analysis of 27 social work supervision studies. They found that supervision was frequently measured by one or two of the following context dimensions: task assistance, emotional support, and supervisory personal interaction. All three dimensions were significantly related to positive job outcomes for social workers [39]. Similarly, Wilkins and Antonopoulou studied 315 social workers from the United Kingdom and found that supervision frequency was positively associated with positive job outcomes [41]. Hung et al. used focus group interviews of social workers in Shenzhen, China and found that respondents felt satisfaction with supervision when the supervisor provided task assistance and emotional support [45]. However, it is important to note that past research on social work supervision has been largely qualitative in nature [40,42,44,48] and is mainly restricted to Western countries’ experiences [40,41,48]. While supervision should have a powerful effect on social workers, it should occur regardless of their geographic location. 

With respect to supervision regulation in social work in China, the Ministry of Civil Affairs issued the Guidelines for Social Work Supervision in 2021 [49]. These guidelines stipulate the area, process, ethics, and deployment of supervision, including supervisor credentials, and implement a cap of 5 supervisees per supervisor. However, the guidelines do not specify the frequency nor duration of supervision. Consequently, local governments and social work associations often issue their own guidelines on supervision frequency and duration [50]. Though varied by cities and provinces, the local guidelines generally recommend one supervision session per month, and the duration of supervision varies with the supervisee’s work experience. For example, the Guangzhou Social Work Association recommends one supervision session per month, which lasts 2 h for social workers with 2 or fewer years of experience, or 1 h for workers with more than 2 years of experience [51].

Social work supervision provides social workers with opportunities to manage their workloads and emotional labor in a safe and supported way [36,37,38]. Additionally, supervision ensures that social workers can continue to provide professional, quality services to their clients [39,40,41]. Moreover, supervision has the potential to reduce work stress and increase job satisfaction amongst social workers [45,46,47,48]. 

This paper seeks to expand the academic understanding of how supervision is performed, and its influence on Chinese social workers. So far, there has been plentiful research documenting the high emotional stress, such as negative affect and psychological distress, that Chinese social workers experience [7,12,52]. However, this study uses empirical data to examine the effects of supervision on negative affect and psychological distress amongst Chinese social workers. These findings can advance the understanding of how negative affect and psychological distress influences Chinese social workers and whether supervision may mitigate any subsequent adverse effects. As Meunier and colleagues [28] note, when adequate supervision occurs, where the supervisor implements strong communication techniques and shows supportive practices to their employees, workers are less likely to feel isolated at work or experience psychological strain, and are overall more likely to have increased well-being. Therefore, we hypothesize that:

**Hypothesis** **1**: *Supervision is negatively associated with negative affect*.

**Hypothesis** **2**: *Supervision moderates the effects of job demands on negative affect*.

**Hypothesis** **3**: *Supervision is negatively associated with psychological distress*.

**Hypothesis** **4**: *Supervision moderates the effects of job demands on psychological distress*.

## 2. Methods

### 2.1. Data and Sample

We utilized an online anonymous survey to collect our data. The survey was originally written in English, but was later translated to Chinese by two Chinese doctoral students in the United States and was further verified by an American professor whose native language is Chinese. The survey and research procedure were approved by the Research Review Committee, School of Public Administration, Guangdong University of Foreign Studies in China on 15 June 2021. On 15 September 2021, we sent the survey link to 756 frontline social workers from 54 randomly selected agencies throughout Guangzhou, China. Thereafter we emailed participants two reminders to participate on 22 September 2021, and on 29 September 2021. Of the 756 frontline social workers emailed, 537 social workers participated by 10 October 2021, resulting in a 71% response rate. After review, we excluded 48 social workers who worked as supervisors within their agencies for our final analysis because we were examining the effects of supervision on supervisees only. Thus, our final sample consisted of 489 frontline social workers. Each respondent was additionally informed of their participation rights and informed of their ability to end the survey at will. The primary demographics of the final sample were majority female (85.1%) and never married (56.4%). The mean age was 29 years old, and more than half of the sample had at least a college degree. 

### 2.2. Measures

First, to measure negative affect we used an abbreviated version of the International Positive and Negative Affect Schedule (I-PANAS-SF) [53]. The I-PANAS-SF has been shown to have cross-sample stability, internal reliability, temporal stability, cross-cultural factorial invariance, and convergent and criterion-related validity, which makes it an effective tool [53,54,55]. Essentially, the scale measured the participants’ emotions, such as hostility and shame, throughout the previous two-week period. Participants were able to select one answer along a 5-point interval scale ranging from 1 (“never”) to 5 (“always”). The responses to each item were then averaged. Each participant could have a final score ranging from 1 to 5, and the higher the score, the more negative affect was present. Here, the Cronbach’s alpha of negative affect was 0.88. 

Second, to measure psychological distress, we used the valid and reliable Kessler 6 Psychological Distress Scale (“K6”) [30,56,57,58]. The K6’s measure of psychological distress has been calibrated and shown reliable through previous studies [29,56,59]. Participants were asked to self-report how often they experienced psychological distress within the past 30-day period. These included feelings of nervousness, hopelessness, restlessness, worthlessness, and/or depression. For example, one of the prompts asked participants to rate how frequently they felt “everything was an effort” [56]. Participants could rate their response ranging along a 5-point scale from 0 (“none of the time”) to 4 (“all of the time”). Next, we added all responses from each item, giving each participant a psychological distress score between 0 and 24. In this study, the K6 scale had a Cronbach’s alpha value of 0.94. 

Third, we followed previous studies [39,41,45] and critiqued the influence of supervision through three lenses: (1) frequency, (2) type, and (3) reported satisfaction in task assistance and emotional support areas. We measured frequency of supervision by asking respondents to report how often they had had a supervision meeting within the last year. Respondents could answer either between “3 times or less”, “4–6 times”, “7–9 times”, or “10 times or more”. Next, we assessed for type of supervision by asking respondents to report what kind of supervision they experienced at work. These options ranged from: “individual”, “group”, or “both individual and group” supervision. Finally, we measured satisfaction with supervision by asking respondents whether they felt satisfied with the help they received in each of the following supervision topics: case management, program design and implementation, resource integration and utilization, professional knowledge, professional ethics, ability to oversee team members, and emotional counseling and support. Respondents could respond either 0 (“not satisfied”) or 1 (“satisfied”). Each of the answers were then summed to get a final score ranging from 0 to 7. 

Fourth, we used Lequeurre et al.’s Questionnaire sur les Ressources et Contraintes Professionnelles (QRCP) [60] to measure job demands. For this assessment, we considered three influencing aspects of Chinese social workers’ job responsibilities: pace and amount of workload given to each worker, emotional workload, and changes within tasks. First, pace and amount of workload examined the quantity of work or tasks that social workers are given in a relatively constrained time. Second, emotional workload examined the quantity of emotional energy that social workers are required to utilize throughout their workdays. Third, changes in tasks examined how changes in job tasks created challenges for workers. Lequeurre and colleagues used 4 prompts to measure each of these three influential aspects of job demands [60]. For example, participants were asked to self-report how often they felt they had “Too much work to do”. Possible answers ranged along a 7-point Likert scale from 1 (“never”) to 7 (“always”). We then averaged the scores from all the items. The higher the score, the more job demands were present. Overall, the Cronbach’s alpha was 0.83 in this study.

The analysis also factored in certain demographics and socioeconomic characteristics from the participants, such as gender (female = 1, male = 0), age, marital status (never married = 1, other = 0), and education (college degree or above = 1, below college education = 0). 

### 2.3. Analytical Approach

To analyze our sample, we used STATA software 16.0 to perform descriptive analyses that illustrate the respondents’ characteristics among all variables and an ordinary least squares (OLS) regression analysis to estimate the effects that supervision had on negative affect and psychological distress. Throughout these analyses, we controlled for socioeconomic characteristics of the respondents to ensure that these factors did not influence our results. Finally, we assessed for a moderation effect by adding the interaction between job demands and supervision into the analysis [61].

## 3. Results

Table 1 demonstrates the variables’ descriptive statistics. Respondents reported, on average, a score of 2.4 for negative affect (SD = 0.9) and 7.5 for psychological distress (SD = 5.4). A majority of the sample also reported having supervision meetings 10 times or more annually (82.0%), followed by three times or less (9.6%), seven to nine times (5.1%), and four to six times annually (3.3%). Additionally, most of the respondents report experiencing both individual and group supervision at work (90.8%), followed by group supervision only (5.7%), and individual supervision only (3.5%). The average satisfaction with supervision was 3.7 (SD = 1.8), ranging from 0 to 7. Respondents also conferred relatively high job demands (M = 4.7, SD = 0.7). Together, these results suggest that, although the sampled social workers had high job demands, most of them also frequently received individual and/or group supervision. Finally, most of the respondents also reported moderate satisfaction with supervision along with modest negative affect and psychological distress. 

Table 2, Table 3 and Table 4 demonstrate the standardized estimates of negative affect and psychological distress, estimated by the OLS regression. Table 2 and Table 3, respectively, focus on frequency and type of supervision, while Table 4 examines the effects of satisfaction with supervision. Moreover, four models were presented in each Table. Model 1 demonstrates the effects of supervision and job demands on negative affect, while Model 2 demonstrates the interaction between supervision and job demands. Models 3 and 4 are respectively the same as Models 1 and 2, except that each uses psychological distress as the dependent variable rather than negative affect. 

As shown in Table 2, the adjusted R-square of Model 1 was 0.18. As expected, supervision and job demands have significant effects on negative affect. Social workers who had 10 or more supervision sessions at work annually, compared to social workers who had supervision 0 to 3 times annually, reported a 0.15-standard-deviation-lower score on negative affect (*p* < 0.01). These results confirm Hypothesis 1: frequent supervision reduces negative affect. Likewise, the data showed that an increase of 1 standard deviation in job demands was associated with an increase of 0.65 standard deviations in negative affect (*p* < 0.001). Additionally, the interaction between category 4 of supervision (10 times or more) and job demands was significant (Beta = −0.71, *p* < 0.05) (See Model 2). This finding supports Hypothesis 2: high frequency of supervision moderates the effect of job demands on negative affect. 

With respect to the results on psychological distress, the adjusted R-square of Model 3 was 0.25. Supervision and job demands also have significant effects on psychological distress. Social workers who underwent supervision 10 times or more annually, compared to social workers who attended supervision zero to three times per year, reported a 0.11-standard-deviation-lower distress level at work (*p* < 0.05). Additionally, our results show that social workers who underwent supervision seven to nine times annually also had significantly low psychological distress (beta = −0.09, *p* < 0.05). These findings confirm Hypothesis 3. Finally, an increase of 1 standard deviation in job demands was associated with an increase of 0.49 standard deviations in psychological distress amongst social workers (*p* < 0.001). 

The results in Model 4 indicate that the interaction between category 4 of supervision (10 times or more annually) and job demands was significant (Beta = −0.72, *p* < 0.05), and similarly for the interaction between category 3 of supervision (7–9 times) and job demands (Beta = −0.54, *p* < 0.05). These findings confirm Hypothesis 4 and suggest that supervision moderates the effects of job demands on psychological distress.

Importantly, the type of supervision showed no effect on negative affect and psychological distress in Models 1 and 3 in Table 3; however, the interaction between the type of supervision and job demands was significant in Models 2 and 4. This significant interaction suggests that when job demands at work are high, social workers who receive both individual and group supervision have significantly lower negative affect and psychological distress as compared to social workers who receive only individual supervision. In addition, social workers who receive only group supervision will tend to have lower psychological distress when job demands are high, compared to social workers who receive only individual supervision. Thus, the type of supervision matters.

Lastly, satisfaction with supervision has significant effects on negative affect and psychological distress, as shown in Table 4. Increasing 1 standard deviation of satisfaction with supervision would lead to 0.09 standard deviations lower of negative affect and 0.08 standard deviations lower of psychological distress. However, the interactions between satisfaction with supervision and job demands were not significant. 

## 4. Discussion

The findings from the regression analysis support our hypotheses that supervision reduces negative affect and psychological distress amongst Chinese social workers. The results are consistent with previous findings that supervision provides emotional support and reduces distress [41,43,44,45,47,48]. The effects were stronger for social workers who had both higher job demands and frequent, rewarding supervision. The estimates suggest that supervision has a relatively large effect when the frequency of supervision is 10 times or more annually. The findings also support the supervision guideline in Guangzhou that specify that supervision should be only as frequent as once per month [51]. It is especially crucial to maintain frequent supervision for social workers with high job demands and who thus may need more emotional support and professional guidance [14,45].

Moreover, the importance of supervision satisfaction is highlighted in this study. It is essential for supervisors to understand the needs of their supervised social workers and to listen to supervisees’ constructive feedback to better provide suitable supervision and sufficient advice [43,45,50]. According to the results of this study, the current level of supervision satisfaction is around the mean. Thus, supervisors need to modify their practices to become more effective, so supervision can further reduce negative affect and psychological distress amongst social workers in China. Finally, when job demands were high, social workers who received both individual and group supervision also appeared to have lower negative affect and psychological distress as compared to social workers who only received individual supervision. Thus, supervisors might continue to use mixed approaches to provide supervision, as social workers could learn and receive support from both supervisors and colleagues. However, supervisors also need to be mindful of Chinese traditional culture, particularly face culture, during group supervision. Face culture refers to the cultural need to avoid criticism and confrontation, especially in group setting. Traditional Chinese values emphasize harmony within interpersonal dynamics, so supervisors and peer supervisees should be mindful to how they deliver constructive feedback and suggestions to supervisees [50,62].

The findings also support our hypotheses that job demands influence both negative affect and psychological distress for Chinese social workers. The average of our sample experienced relatively high job demands at work. Thus, it is critical that social work employers be aware and cautious of placing high job demands onto their employees because it can lead to negative mental health outcomes that increase burnout and turnover throughout the agency [13,23,30,32]. This is particularly relevant as the job responsibilities of Chinese social workers tend to be wide-ranging and demanding [14,15,18,19]. Employers should implement interventions that seek to reduce negative affect and psychological distress. For instance, studies on mindfulness-based interventions have provided evidence of the effectiveness of mindfulness on improving positive affect and reducing negative affect [63,64,65,66,67]. It may be financially more difficult for smaller agencies to take on these efforts, so policymakers should consider requiring the federal government to subsidize funding and aid to ensure that all social workers are protected equally regardless of access or means.

However, our results do have some limitations. For example, the associative relationship between supervision, negative affect, and psychologic distress could only be approximated because the analysis was based on a cross-sectional dataset. Future studies might prefer to use a longitudinal design to better approximate the causal relationships of these variables. Additionally, there were certain unobserved variables that were likely excluded from the study, such as personality traits of the participants, years of professional experience, training of supervisors, models of supervision, and other contextual characteristics [44]. These excluded variables may have had effects on the relationship between supervision, negative affect, and psychologic distress that went unaccounted for. Moreover, our data was collected using a self-reporting survey. This means the participants may have intentionally or unintentionally overreported/underreported certain aspects of their experience when it came to supervision, negative affect, and psychologic distress. Future research might consider data triangulation through colleague and employer reports instead of this method. Last, the results are narrowly focused on the experience of social workers from Guangzhou, a major city in China. Even though the results were well supported, it is unknown how much they apply to social workers across China in different regions. Thus, further investigation is required.

## 5. Conclusions

This study utilized data from 489 social workers in Guangzhou, China, to understand if there was a relationship between supervision, job demands, negative affect, and psychologic distress amongst Chinese social workers. Our results agreed with past findings from cross-cultural research, which have indicated that supervision reduces negative affect and psychological distress, while job demands increase them. The findings emphasize that supervision has a significant influence on Chinese social workers: supervision is a buffer factor to reduce negative affect and psychological distress for social workers who face high job demands.

## Figures and Tables

**Table 1:** Descriptive statistics of key variables.

	Mean (S.D.)
1. Negative Affect (1–5)	2.4 (0.9)
2. Psychological Distress (0–24)	7.5 (5.4)
3. Frequency of Supervision (%)	
≤3	9.6
4–6	3.3
7–9	5.1
≥10	82.0
4. Type of Supervision (%)	
Individual	3.5
Group	5.7
Individual and Group	90.8
5. Satisfaction w. Supervision (0–7)	3.7 (1.8)
6. Job Demand (1–7)	4.7 (0.7)
7. Female (%)	85.1
8. Age (18–60)	29.0 (6.4)
9. Education (%)	
Below College	49.5
College and Above	50.5
10. Marital Status (%)	
Never Married	56.4
Married	44.6

Note: N = 489. Numbers in brackets show ranges of the variables.

**Table 2:** Regression analysis of negative affect and psychological distress by frequency of supervision.

	Negative Affect						Psychological Distress					
	Model 1			Model 2			Model 3			Model 4		
	Beta	S. E.	*p*	Beta	S. E.	*p*	Beta	S. E.	*p*	Beta	S. E.	*p*
Supervision												
(1): ≤3	-	-		-	-		-	-		-	-	
(2): 4–6	0.01	0.23		0.39	1.39		−0.03	1.36		0.21	8.37	
(3): 7–9	−0.07	0.19		0.40	1.11		−0.09	1.16	*	0.43	6.69	
(4): ≥10	−0.15	0.12	**	0.49	0.68		−0.11	0.73	*	0.53	4.07	
Job Demands	0.42	0.05	***	0.65	0.13	***	0.49	0.30	***	0.72	0.82	***
Supervision * Job Demands												
(1) * Job Demands	-	-		-	-		-	-		-	-	
(2) * Job Demands	-	-		−0.39	0.31		-	-		−0.25	1.84	
(3) * Job Demands	-	-		−0.48	0.24		-	-		−0.54	1.45	*
(4) * Job Demands	-	-		−0.71	0.15	*	-	-		−0.72	0.89	*
Female	−0.07	0.10		−0.07	0.10		−0.06	0.61		−0.06	0.61	
Age	−0.02	0.01		0.02	0.01		−0.08	0.04		−0.08	0.04	
Education—College and Above	−0.02	0.07		−0.03	0.07		0.00	0.43		0.00	0.43	
Never Married	0.06	0.09		0.06	0.09		0.06	0.54		0.06	0.54	
Adjusted R-square	0.18			0.19			0.25			0.26		

Note: N = 489. * *p* < 0.05, ** *p* < 0.01, *** *p* < 0.001.

**Table 3:** Regression analysis of negative affect and psychological distress by type of supervision.

	Negative Affect						Psychological Distress					
	Model 1			Model 2			Model 3			Model 4		
	Beta	S. E.	*p*	Beta	S. E.	*p*	Beta	S. E.	*p*	Beta	S. E.	*p*
Type of Supervision												
(1): Individual	-	-		-	-		-	-		-	-	
(2): Group	−0.06	0.24		0.73	1.53		0.02	1.46		0.82	9.18	*
(3): Individual and Group	−0.01	0.20		0.86	1.27	*	0.04	1.17		1.02	7.65	*
Job Demands	0.40	0.05	***	0.82	0.25	***	0.48	0.31	***	0.95	1.51	***
Type of Supervision * Job Demands												
(1) * Job Demands	-	-		-	-		-	-		-	-	
(2) * Job Demands	-	-		−0.72	0.33		-	-		−0.72	1.95	*
(3) * Job Demands	-	-		−0.94	0.26	*	-	-		−1.05	1.55	*
Female	−0.08	0.10		−0.06	0.10		−0.07	0.61		−0.05	0.61	
Age	−0.02	0.01		−0.02	0.01		−0.08	0.04		−0.08	0.04	
Education—College and Above	−0.03	0.07		−0.03	0.07		0.00	0.43		0.00	0.43	
Never Married	0.06	0.09		0.07	0.09		0.06	0.54		0.06	0.54	
Adjusted R-square	0.17			0.18			0.25			0.25		

Note: N = 489. * *p* < 0.05, *** *p* < 0.001.

**Table 4:** Regression analysis of negative affect and psychological distress by satisfaction with supervision.

	Negative Affect						Psychological Distress					
	Model 1			Model 2			Model 3			Model 4		
	Beta	S. E.	*p*	Beta	S. E.	*p*	Beta	S. E.	*p*	Beta	S. E.	*p*
Satisfaction w. Supervision	−0.09	0.02	*	−0.14	0.13		−0.08	0.12	*	0.26	0.75	
Job Demands	0.42	0.05	***	0.40	4.81	***	0.49	0.30	***	0.59	0.61	***
Satisfaction w. Supervision * Job Demands				0.05	0.03					−0.37	0.15	
Female	−0.08	0.10		−0.08	0.10		−0.06	0.60		−0.06	0.60	
Age	−0.03	0.01		−0.03	0.01		−0.09	0.04		−0.09	0.04	
Education—College and Above	−0.02	0.07		−0.02	0.07		0.00	0.43		0.00	0.43	
Never Married	0.06	0.09		0.06	0.09		0.06	0.54		0.06	0.54	
Adjusted R-square	0.18			0.18			0.26			0.26		

Note: N = 489. * *p* < 0.05, *** *p* < 0.001.

## Data Availability

Data available on request due to privacy restrictions.
